# Influenza, Tdap, and COVID-19 Vaccination Coverage and Hesitancy Among Pregnant Women — United States, April 2023

**DOI:** 10.15585/mmwr.mm7239a4

**Published:** 2023-09-29

**Authors:** Hilda Razzaghi, Katherine E. Kahn, Kayla Calhoun, Emma Garacci, Tami H. Skoff, Sascha R. Ellington, Tara C. Jatlaoui, Carla L. Black

**Affiliations:** ^1^Immunization Services Division, National Center for Immunization and Respiratory Diseases, CDC; ^2^Leidos, Atlanta, Georgia; ^3^Cherokee Nation Operational Solutions, Tulsa, Oklahoma; ^4^Division of Bacterial Diseases, National Center for Immunization and Respiratory Diseases, CDC; ^5^Influenza Division, National Center for Immunization and Respiratory Diseases, CDC.

**Summary**

**What is already known about this topic?**

Influenza, tetanus toxoid, reduced diphtheria toxoid, and acellular pertussis (Tdap), and COVID-19 vaccines can reduce the risk for severe respiratory illness among pregnant women and their infants.

**What is added by this report?**

During the 2022–23 influenza season, 47.2% of women received influenza vaccination before or during pregnancy, 55.4% of women with a recent live birth received Tdap vaccination during pregnancy, and 27.3% of women received a COVID-19 bivalent booster vaccine before or during pregnancy. Pregnant women who received a provider recommendation for vaccination were less hesitant about influenza and Tdap vaccines.

**What are the implications for public health practice?**

Promotion of efforts to improve vaccination coverage among pregnant women, such as provider recommendation for vaccination and informative conversations with patients to address vaccine hesitancy, could reduce adverse maternal and infant illness and death from vaccine-preventable diseases.

## Abstract

Influenza, tetanus toxoid, reduced diphtheria toxoid, and acellular pertussis (Tdap), and COVID-19 vaccines can reduce the risk for influenza, pertussis, and COVID-19 among pregnant women and their infants. To assess influenza, Tdap, and COVID-19 vaccination coverage among women pregnant during the 2022–23 influenza season, CDC analyzed data from an Internet panel survey conducted during March 28–April 16, 2023. Among 1,814 survey respondents who were pregnant at any time during October 2022–January 2023, 47.2% reported receiving influenza vaccine before or during their pregnancy. Among 776 respondents with a live birth by their survey date, 55.4% reported receiving Tdap vaccine during pregnancy. Among 1,252 women pregnant at the time of the survey, 27.3% reported receipt of a COVID-19 bivalent booster dose before or during the current pregnancy. Data from the same questions included in surveys conducted during influenza seasons 2019–20 through 2022–23 show that the proportion of pregnant women who reported being very hesitant about influenza and Tdap vaccinations during pregnancy increased from 2019–20 to 2022–23. Pregnant women who received a provider recommendation for vaccination were less hesitant about influenza and Tdap vaccines. Promotion of efforts to improve vaccination coverage among pregnant women, such as provider recommendation for vaccination and informative conversations with patients to address vaccine hesitancy, might reduce vaccine hesitancy and increase coverage with these important vaccines to protect mothers and their infants against severe respiratory diseases.

## Introduction

Maternal vaccination with influenza vaccine and tetanus toxoid, reduced diphtheria toxoid, and acellular pertussis (Tdap) vaccine during pregnancy can reduce the risk for influenza and pertussis among pregnant women and their infants. The Advisory Committee on Immunization Practices (ACIP) recommends that all women who are or might be pregnant during the influenza season receive influenza vaccine, which can be administered at any time during pregnancy (*1*). ACIP also recommends that women receive Tdap vaccine during each pregnancy, preferably early during the period from 27 to 36 weeks’ gestation (*2*,*3*). In addition, COVID-19 vaccines are recommended for all persons aged ≥6 months,* including women who are pregnant.^†^ Despite recommendations for vaccination, coverage during pregnancy with all three vaccines is low and varies by certain characteristics as well as provider recommendation and offer of vaccination during a visit or referral to a vaccine provider (*4*,*5*).

## Methods

An Internet panel^§^ survey was conducted to assess end-of-season influenza and Tdap vaccination coverage estimates among women who were pregnant during the 2022–23 influenza season, as previously described (*4*). The survey was conducted during March 28–April 16, 2023, among women aged 18–49 years who reported being pregnant at any time since August 1, 2022, through the date of the survey. Among 17,931 women who entered the survey site and answered the screening questions, 2,588 were eligible, and of these, 2,349 (90.8%) completed the survey.^¶^ Data were weighted to reflect pregnancy status and outcome at the time of survey completion, age, race and ethnicity, and geographic distribution of the total U.S. population of pregnant women.

Analysis of influenza vaccination coverage was limited to 1,814 women who reported being pregnant at any time during October 2022–January 2023. A woman was considered to have been vaccinated against influenza if she reported receiving a dose of influenza vaccine (before or during her most recent pregnancy) since July 1, 2022. To accommodate the optimal timing for Tdap vaccination during gestational weeks 27–36, analysis of Tdap vaccination coverage was limited to women who reported having been pregnant at any time since August 1, 2022, and who had a live birth by their survey date. A woman was considered vaccinated with Tdap if she reported receiving a dose of Tdap vaccine during her most recent pregnancy. Among 890 women with a recent live birth, 114 (12.8%) were excluded because they did not know if they had ever received Tdap vaccine (88; 9.9%) or if Tdap vaccine was received during their pregnancy (26; 2.9%), leaving a final analytic sample of 776. The proportion of pregnant women who had received both recommended maternal vaccines was assessed among 775 women (one respondent who was excluded reported her Tdap vaccination status [not vaccinated], but not her influenza vaccination status).

COVID-19 vaccination coverage was assessed among 1,252 women who were pregnant at the time of the survey. COVID-19 vaccination coverage was assessed on the basis of receipt of ≥1 dose,** completion of a primary series,^††^ and receipt of a bivalent booster dose^§§^ before or during the current pregnancy, according to ACIP recommendations at the time of the survey (*6*). To assess changes in influenza and Tdap-specific vaccine hesitancy among pregnant women over time, data from the same questions included in surveys conducted during influenza seasons 2019–20 through 2022–23 were used. SAS-callable SUDAAN software (version 11.0.1; RTI International) was used to conduct all analyses. Differences among groups were assessed using t-tests with p-values <0.05 considered statistically significant. All reported increases or decreases are statistically significant. This activity was reviewed by CDC, deemed research not involving human subjects, and was conducted consistent with applicable federal law and CDC policy.^¶¶^

## Results

Among 1,814 women pregnant during October 2022–January 2023, 47.2% reported receiving an influenza vaccination since July 1, 2022 (Table 1); Tdap vaccination coverage during pregnancy was 55.4% among women with a recent live birth. Receipt of both influenza and Tdap vaccines was reported by 25.6% of women with a recent live birth. Vaccination coverage with Tdap alone and both influenza and Tdap vaccines was lower among non-Hispanic Black or African American (Black) women (31.4% and 12.0%, respectively) than among non-Hispanic White (White) women (62.2% and 26.6%, respectively). Tdap vaccination coverage was also lower among Hispanic or Latino (Hispanic) women (50.8%) compared with that among White women (62.2%).

**TABLE 1:** Influenza* vaccination and tetanus toxoid, reduced diphtheria toxoid, and acellular pertussis vaccination^†^ coverage among pregnant women, by selected characteristics — Internet panel survey, United States, April 2023

Characteristic	Influenza vaccine		Tdap vaccine		Both influenza and Tdap vaccines	
	Total no. (weighted %)^§^	Weighted % vaccinated (95% CI)^¶^	Total no. (weighted %)^§^	Weighted % vaccinated (95% CI)^¶^	Total no. (weighted %)^§^	Weighted % vaccinated (95% CI)^¶^
**Overall**	**1,814 (100.0)**	**47.2 (44.4–50.1)**	**776 (100.0)**	**55.4 (51.5–59.3)**	**775 (100.0)**	**25.6 (22.1–29.3)**
**Age group, yrs**						
18–24	**329 (23.3)**	39.9 (33.0–47.0)**	121 (20.1)	51.6 (41.4–61.7)	120 (20.0)	20.7 (12.5–31.1)**
25–34	**916 (57.3)**	48.9 (45.1–52.7)	442 (60.8)	55.9 (50.7–61.1)	442 (60.9)	25.0 (20.5–30.0)
35–49 (Ref)	**569 (19.4)**	51.3 (46.5–56.0)	213 (19.1)	58.0 (50.7–64.9)	213 (19.2)	32.5 (25.9–39.6)
**Race and ethnicity^††^**						
Black or African American	**227 (16.6)**	39.5 (31.0–48.5)	88 (14.5)	31.4 (21.5–42.7)**	87 (14.3)	12.0 (5.8–21.1)**
White (Ref)	**1,011 (50.5)**	46.1 (42.5–49.8)	502 (54.1)	62.2 (57.8–66.5)	502 (54.2)	26.6 (22.7–30.8)
Hispanic or Latino	**427 (23.3)**	52.7 (47.0–58.3)	131 (22.7)	50.8 (41.1–60.5)**	131 (22.7)	23.8 (15.8–33.4)
Other	**149 (9.7)**	53.2 (42.6–63.6)	55 (8.7)	—^§§^	55 (8.7)	—^§§^
**Education**						
High school diploma or less	**506 (31.4)**	36.2 (31.0–41.8)**	220 (29.2)	45.7 (38.7–52.9)**	219 (29.1)	15.0 (10.4–20.6)**
Some college, no degree	**388 (22.1)**	40.6 (34.8–46.5)**	163 (22.4)	60.6 (51.9–68.8)	163 (22.4)	30.4 (22.2–39.6)
College degree	**634 (33.9)**	54.3 (49.1–59.4)**	293 (36.6)	58.2 (51.4–64.8)	293 (36.7)	27.9 (22.2–34.3)
Higher than college degree (Ref)	**286 (12.6)**	67.5 (60.7–73.7)	100 (11.8)	61.2(49.1–72.3)	100 (11.8)	35.5 (24.1–48.2)
**Employment status**						
Working (Ref)	**1,253 (67.4)**	52.7 (49.3–56.1)	477 (61.8)	53.0 (47.9–58.1)	476 (61.7)	26.0 (21.4–31.0)
Not working	**560 (32.6)**	35.9 (30.9–41.0)**	299 (38.2)	59.3 (52.9–65.5)	299 (38.3)	25.0 (19.7–30.9)
**Poverty status** ^¶¶^						
At or above poverty level (Ref)	**1,325 (69.7)**	54.3 (51.0–57.5)	571 (72.5)	57.7 (53.0–62.3)	571 (72.6)	28.2 (24.0–32.7)
Below poverty level	**480 (30.3)**	31.4 (26.4–36.8)**	205 (27.5)	49.5 (41.9–57.2)	204 (27.4)	18.6 (12.8–25.8)**
Area of residence***						
Rural	**361 (18.0)**	38.4 (32.7–44.5)**	189 (22.9)	59.3 (51.3–67.0)	189 (22.9)	21.9 (15.2–29.9)
Nonrural (Ref)	**1,453 (82.0)**	49.2 (45.9–52.4)	587 (77.1)	54.3 (49.7–58.8)	586 (77.1)	26.7 (22.7–31.0)
**U.S. Census Bureau region^†††^**						
Northeast (Ref)	**280 (17.6)**	52.3 (44.5–60.0)	109 (16.0)	56.3 (45.6–66.6)	108 (15.9)	31.1 (21.4–42.3)
Midwest	**402 (20.1)**	47.6 (41.5–53.8)	181 (21.1)	60.9 (52.9–68.6)	181 (21.1)	26.2 (19.9–33.3)
South	**745 (38.3)**	44.0 (39.5–48.6)	337 (39.9)	54.1 (48.3–59.9)	337 (40.0)	21.2 (16.8–26.2)
West	**387 (24.1)**	48.4 (42.2–54.7)	149 (23.0)	52.1 (42.3–61.9)	149 (23.0)	28.9 (19.9–39.3)
**Prenatal insurance coverage^§§§^**						
Private or military insurance only (Ref)	**761 (39.6)**	54.9 (50.5–59.2)	372 (45.2)	61.4 (55.7–66.8)	372 (45.3)	33.7 (28.2–39.5)
Any public insurance	**985 (56.1)**	42.7 (38.7–46.7)**	385 (52.3)	50.8 (45.2–56.5)**	384 (52.3)	19.4 (15.0–24.4)**
No insurance	**68 (4.3)**	—^§§^	19 (2.5)	—^§§^	19 (2.5)	—^§§^
**Provider vaccination recommendation or offer** ^¶¶¶^						
Offered or referred (Ref)	**1,306 (70.9)**	61.4 (58.0–64.7)	620 (79.2)	69.1 (65.0–73.0)	486 (63.5)****	37.5 (32.7–42.4)
Recommended, no offer or referral	**125 (7.6)**	22.7 (15.0–32.0)**	23 (3.8)	—^§§^	230 (29.7)^††††^	5.0 (2.5–8.9)**
No recommendation	**356 (21.5)**	10.8 (7.5–14.9)**	133 (17.0)	—^§§^	49 (6.8) ^§§§§^	0 (0–7.3)**
**No. of provider visits since Jul 1, 2022**						
None	**26 (1.9)**	—^§§^	NA	NA	NA	NA
1–5	**704 (41.2)**	45.2 (40.6–49.8)	NA	NA	NA	NA
6–10	**442 (24.2)**	52.5 (46.3–58.6)	NA	NA	NA	NA
>10 (Ref)	**640 (32.7)**	46.9 (42.2–51.6)	NA	NA	NA	NA
**High-risk condition for influenza** ^¶¶¶¶^						
Yes (Ref)	**837 (48.4)**	49.3 (45.2–53.5)	NA	NA	NA	NA
No	**899 (51.6)**	44.4 (40.5–48.3)	NA	NA	NA	NA

**Abbreviations:** NA = not applicable; Ref = referent group; Tdap = tetanus toxoid, reduced diphtheria toxoid, and acellular pertussis.

* Respondents pregnant at any time during October 2022–January 2023 were included in the analyses to assess influenza vaccination coverage for the 2022–23 season. Women who reported receiving an influenza vaccination since July 1, 2022, before or during their pregnancy, were considered vaccinated.

^†^ Respondents pregnant since August 1, 2022, with a recent live birth were included in the analyses to assess Tdap vaccination coverage. Women who reported receiving a Tdap vaccination during their pregnancy were considered vaccinated.

^§^ The total unweighted number and weighted proportion of respondents in the sample.

^¶^ Korn-Graubard 95% CI.

** Statistically significant difference compared with Ref.

^††^ Race and ethnicity were self-reported. Persons of Hispanic or Latino (Hispanic) origin might be of any race but are categorized as Hispanic; all racial groups are non-Hispanic. The “Other” race category included Asian, American Indian or Alaska Native, Native Hawaiian or other Pacific Islander, and women who selected multiple races.

^§§^ Dashes indicate estimates do not meet the National Center for Health Statistics’ standards of reliability. https://www.cdc.gov/nchs/data/series/sr_02/sr02_175.pdf

^¶¶^ Poverty status was defined on the basis of the reported number of persons living in the household and annual household income, according to U.S. Census Bureau poverty thresholds. https://www.census.gov/data/tables/time-series/demo/income-poverty/historical-poverty-thresholds.html

*** Rurality was defined using zip codes where >50% of the population lives in a nonmetropolitan county, a rural U.S. Census Bureau tract, or both, according to the Health Resources and Services Administration’s definition. https://www.hrsa.gov/rural-health/about-us/what-is-rural

^†††^ https://www2.census.gov/geo/pdfs/maps-data/maps/reference/us_regdiv.pdf

^§§§^ Respondents pregnant on their survey date were asked what medical insurance or medical care coverage they had; respondents who had already delivered were asked what coverage they had during their most recent pregnancy. Women considered to have public insurance selected at least one of the following: Medicaid, Medicare, state-sponsored medical plan, or other government plan. Respondents considered to have private or military insurance selected private medical insurance or military medical care and did not select any type of public insurance.

^¶¶¶^ Excluded women from influenza vaccination analyses who did not report having a provider visit since July 2022 (27).

**** Received provider offer or referral for both influenza and Tdap vaccines.

^††††^ Received a combination of provider offer or referral, recommendation with no referral, or no recommendation for influenza or Tdap vaccines that did not include receipt of offer or referral for both vaccines or no recommendation received for both vaccines. For example, the respondent might have received an offer or referral for influenza vaccine and a recommendation with no referral for Tdap vaccine.

^§§§§^ Did not receive a provider recommendation for influenza or Tdap vaccines.

^¶¶¶¶^ Conditions other than pregnancy associated with increased risk for serious medical complications of influenza include chronic asthma, a lung condition other than asthma, a heart condition, diabetes, a kidney condition, a liver condition, obesity, sickle cell disease, a neurologic or neuromuscular condition, or a weakened immune system caused by a chronic illness or by medicines taken for a chronic illness. Women who were missing information were excluded from analysis (78).

Influenza vaccination coverage was higher among women who reported receiving a provider offer for vaccination during a visit or a referral to a vaccine provider (61.4%) than among those who received a vaccination recommendation but no offer or referral (22.7%) or who received no recommendation (10.8%). Tdap vaccination coverage was similarly high among women who received an offer or referral (69.1%). Influenza vaccination coverage was lower among women living in rural areas, and both influenza and Tdap vaccination coverage were lower among women with public insurance.

Among 1,252 women who were pregnant at the time of the survey, 64.9% reported having received ≥1 COVID-19 vaccine dose, 58.7% reported having completed the primary COVID-19 vaccination series, and 27.3% reported having received a bivalent COVID-19 booster dose (Table 2). Bivalent booster vaccination coverage among women who received a provider recommendation for a bivalent booster (63.2%) was more than nine times that among those who did not (6.8%). Overall, the majority of women who received a bivalent booster dose reported receiving it before their current pregnancy (73.3%).

**TABLE 2:** COVID-19 vaccination coverage among pregnant women, by selected characteristics — Internet panel survey, United States, April 2023

Characteristic	Total no. (weighted %)^†^	Weighted % (95% CI)*		
		Received ≥1 COVID-19 vaccine dose^§^	Completed primary COVID-19 vaccination series^¶^	Received a COVID-19 bivalent booster dose**
**Overall**	**1,252 (100.0)**	**64.9 (61.9–67.8)**	**58.7 (55.6–61.7)**	**27.3 (24.7–30.0)**
**Age group, yrs**				
18–24	**240 (21.2)**	56.7 (49.8–63.4)^††^	48.1 (41.2–55.0)^††^	20.5 (15.3–26.4)^††^
25–34	**590 (58.5)**	65.1 (60.9–69.1)^††^	59.2 (54.9–63.3)^††^	26.2 (22.5–30.0)^††^
35–49 (Ref)	**422 (20.2)**	72.7 (68.0–77.1)	68.5 (63.6–73.1)	37.9 (33.0–43.0)
**Race and ethnicity^§§^**				
Black or African American	**171 (15.3)**	62.8 (54.4–70.7)	53.1 (44.7–61.3)	18.8 (12.9–26.0)^††^
White (Ref)	**647 (51.4)**	63.1 (58.9–67.1)	57.1 (52.8–61.3)	28.4 (24.8–32.2)
Hispanic or Latino	**338 (23.9)**	68.4 (62.8–73.6)	63.2 (57.5–68.6)	31.0 (26.0–36.4)
Other	**96 (9.4)**	69.0 (56.9–79.5)	65.1 (52.8–76.1)	25.9 (16.0–38.0)
**Education**				
High school diploma or less	**332 (28.2)**	47.7 (41.7–53.7)^††^	39.9 (34.0–45.9)^††^	18.7 (14.4–23.8)^††^
Some college, no degree	**269 (22.5)**	55.8 (49.1–62.3)^††^	46.9 (40.2–53.7)^††^	13.8 (9.3–19.3)^††^
College degree	**415 (32.9)**	76.4 (71.5–80.8)	71.6 (66.6–76.3)^††^	31.2 (26.5–36.2)^††^
Higher than college degree (Ref)	**236 (16.5)**	83.6 (77.0–89.0)	81.1 (74.2–86.8)	52.6 (45.3–59.9)
Employment status***				
Working (Ref)	**930 (73.3)**	70.4 (67.0–73.6)	64.3 (60.8–67.7)	32.6 (29.4–35.9)
Not working	**321 (26.7)**	49.7 (43.4–55.9)^††^	43.1 (37.0–49.5)^††^	13.0 (9.1–17.7)^††^
**Poverty status^†††^**				
At or above poverty level (Ref)	**944 (74.5)**	70.8 (67.5–74.0)	65.6 (62.1–68.9)	31.8 (28.6–35.1)
Below poverty level	**298 (25.5)**	47.7 (41.4–54.1)^††^	39.3 (33.1–45.7)^††^	14.0 (10.0–18.9)^††^
**Area of residence^§§§^**				
Rural	**218 (17.2)**	45.9 (38.6–53.3)^††^	40.0 (32.9–47.3)^††^	18.7 (13.6–24.7)^††^
Nonrural (Ref)	**1,034 (82.8)**	68.8 (65.6–71.9)	62.6 (59.2–65.8)	29.1 (26.2–32.2)
**U.S. Census Bureau region^¶¶¶^**				
Northeast (Ref)	**201 (16.8)**	78.1 (70.5–84.5)	72.4 (64.5–79.5)	34.1 (27.1–41.6)
Midwest	**267 (20.7)**	55.6 (48.9–62.1)^††^	49.6 (43.0–56.2)^††^	26.2 (20.7–32.3)
South	**503 (39.1)**	60.8 (55.9–65.5)^††^	53.2 (48.3–58.1)^††^	22.2 (18.6–26.3)^††^
West	**281 (23.4)**	70.4 (64.2–76.0)	66.1 (59.7–72.0)	31.9 (25.9–38.4)
Prenatal insurance coverage****				
Private or military insurance only (Ref)	**506 (40.6)**	76.6 (72.2–80.7)	73.4 (68.8–77.6)	32.3 (28.0–36.9)
Any public insurance	**698 (55.6)**	57.4 (53.3–61.4)^††^	49.4 (45.2–53.6)^††^	25.0 (21.6–28.6)^††^
No insurance	**48 (3.8)**	—^¶¶^	—^¶¶^	—^¶¶^
**Provider recommendation for bivalent booster dose^††††^**				
Yes (Ref)	**529 (62.7)**	NA	NA	63.2 (58.4–67.8)
No	**294 (37.3)**	NA	NA	6.8 (4.0–10.8)^††^
**Timing of receipt of a bivalent booster dose**				
Before current pregnancy	**270 (73.3)**	NA	NA	NA
During current pregnancy	**96 (24.7)**	NA	NA	NA
First trimester	**—^§§§§^ (9.9)**	NA	NA	NA
Second trimester	**—^§§§§^ (13.5)**	NA	NA	NA
Third trimester	**—^§§§§^ (1.3)**	NA	NA	NA

**Abbreviations**: NA = not applicable; Ref = referent group.

* Korn-Graubard 95% CI.

^†^ The total unweighted number and weighted proportion of respondents in the sample.

^§^ Respondents who reported being pregnant at the time of the survey were included in the analysis. Those who reported receiving ≥1 dose of a COVID-19 vaccine before or during their current pregnancy were considered vaccinated.

^¶^ Respondents who reported being pregnant at the time of the survey were included in the analysis. Those who received ≥2 doses of a 2-dose vaccine series (i.e., Pfizer-BioNTech, Moderna, Novavax, or other brand that requires 2 doses) or 1 dose of a 1-dose vaccine (i.e., Janssen [Johnson & Johnson], which requires 1 dose) were considered to have completed the primary series of COVID-19 vaccine; if a respondent reported being immunocompromised (weakened immune system from solid organ transplant, blood or bone marrow transplant, immune deficiencies, HIV, use of corticosteroids, or use of other immune-weakening medicines), an additional dose of a COVID-19 vaccine was required for completion of primary series (66).

** Respondents who reported being pregnant at the time of the survey were included in the COVID-19 bivalent booster analysis. Respondents were considered to have received a COVID-19 bivalent booster vaccine if they responded “yes” to the following question, “Have you received a bivalent booster vaccine*?*” which was asked of women who reported receipt of ≥1 dose of a COVID-19 vaccine.

^††^ Statistically significant difference compared with Ref.

^§§^ Race and ethnicity were self-reported. Persons of Hispanic or Latino (Hispanic) origin might be of any race but are categorized as Hispanic; all racial groups are non-Hispanic. The “Other” race category included Asian, American Indian or Alaska Native, Native Hawaiian or other Pacific Islander, and women who selected multiple races.

^¶¶^ Dashes indicate estimates do not meet the National Center for Health Statistics’ standards of reliability. https://www.cdc.gov/nchs/data/series/sr_02/sr02_175.pdf

*** Respondents were asked about their current work and volunteer activities. Those who reported being a health care worker working directly or not working directly with patients, frontline essential worker (not in health care), essential worker (not in health care and not frontline), or nonessential worker or volunteer were considered to be working. Respondents who indicated that they were not currently working or volunteering were considered to be not working.

^†††^ Poverty status was defined on the basis of the reported number of persons living in the household and annual household income, according to U.S. Census Bureau poverty thresholds. https://www.census.gov/data/tables/time-series/demo/income-poverty/historical-poverty-thresholds.html

^§§§^ Rurality was defined using zip codes where >50% of the population lives in a nonmetropolitan county, a rural U.S. Census Bureau tract, or both, according to the Health Resources and Services Administration’s definition. https://www.hrsa.gov/rural-health/about-us/what-is-rural

^¶¶¶^ https://www2.census.gov/geo/pdfs/maps-data/maps/reference/us_regdiv.pdf

**** Respondents pregnant on their survey date were asked what medical insurance or medical care coverage they had; respondents who had already delivered were asked what coverage they had during their most recent pregnancy. Women considered to have public insurance selected at least one of the following: Medicaid, Medicare, state-sponsored medical plan, or other government plan. Respondents considered to have private or military insurance selected private medical insurance or military medical care and did not select any type of public insurance.

^††††^ Respondents were asked the question, “An updated COVID-19 booster vaccine became available in September 2022 that is known as a ‘bivalent’ booster. It can better protect against the more recent Omicron subvariants as well as the original COVID-19 virus. Has a doctor, nurse, or other health professional ever recommended that you get a COVID-19 bivalent booster?”

^§§§§^ Suppressed to avoid risk of disclosure.

The proportion of respondents who reported being very hesitant about receiving influenza and Tdap vaccines during pregnancy increased significantly during 2022–23 compared with 2019–20. During 2022–23, nearly one quarter (24.7%) of women reported being very hesitant about influenza vaccination during pregnancy compared with 17.2% during 2021–22 and 17.5% during 2019–20. During 2022–23, approximately one in five (19.8%) women reported being very hesitant about Tdap vaccination during pregnancy compared with 14.7% during 2021–22 and 15.1% during 2019–20 (Figure). Hesitancy about influenza and Tdap vaccination has increased since 2019–20 in most demographic subgroups, but remains lower among women who received a provider recommendation for vaccination (Supplementary Table, https://stacks.cdc.gov/view/cdc/132911).

**FIGURE:**
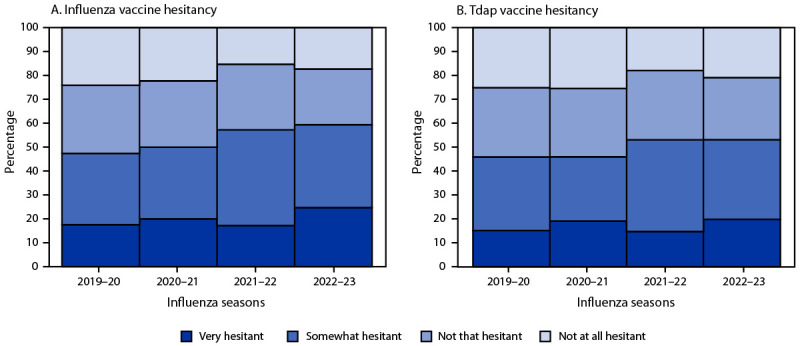
Percentage of pregnant women* who were hesitant^†^ about receiving influenza vaccine (A) and tetanus toxoid, reduced diphtheria toxoid, and acellular pertussis vaccine (B) — Internet panel survey, United States, 2019–20 through 2022–23 influenza seasons **Abbreviation:** Tdap = tetanus toxoid, reduced diphtheria toxoid, and acellular pertussis. * Respondents who were pregnant at any time since August 1 and answered the hesitancy questions (2019–20: 2,261 [influenza and Tdap vaccines]; 2020–21: 2,287 [influenza vaccine] and 2,286 [Tdap vaccine]; 2021–22: 2,485 [influenza vaccine] and 2,484 [Tdap vaccine]; 2022–23: 2,327 [influenza vaccine] and 2,328 [Tdap vaccine]). ^†^ Respondents were asked the following questions, “Overall, how hesitant are you about flu vaccination during your pregnancy?” and “Overall, how hesitant are you about Tdap vaccination during your pregnancy?“ Answer choices were 1) Not at all hesitant, 2) Not that hesitant, 3) Somewhat hesitant, and 4) Very hesitant.

## Discussion

Findings from this survey indicate that approximately one half of pregnant women have not received influenza or Tdap vaccines, and only one quarter received both vaccines, thereby leaving themselves and their infants vulnerable to influenza and pertussis infection. Influenza vaccination coverage remains low and is >10 percentage points (*7*) lower than during the 2019–20 season, consistent with other data sources that have shown decreases in influenza vaccination coverage among pregnant women since the COVID-19 pandemic.*** Although Tdap vaccination coverage increased by approximately 10 percentage points compared with the previous season, coverage during the 2022–23 season is similar to that during the 2019–20 and 2020–21 seasons (*7*,*8*). Approximately three quarters of pregnant women reported not receiving a bivalent COVID-19 booster dose, which might increase the risk for severe COVID-19 disease and pregnancy complications, including hospitalization and death.^†††^

Among pregnant women, influenza, Tdap, and bivalent COVID-19 booster dose coverage remains lower among Black women and those who did not receive a provider recommendation and an offer or referral for vaccination (*5*,*7*–*10*). Studies have noted that a lower percentage of pregnant Black women receive a provider vaccination offer or referral than do women from other racial and ethnic groups (*7*,*10*). The current analysis also found that among pregnant women, influenza and Tdap vaccine hesitancy is higher among Black women compared with White women. A separate analysis found that vaccine hesitancy is associated with lower vaccination coverage; however, a higher percentage of pregnant women who were hesitant about influenza vaccination reported being vaccinated if they received a provider offer or referral for vaccination.^§§§^

These findings along with those from other studies underscore the importance of the equitable provision of provider recommendation and offer or referral for vaccination, in combination with culturally relevant conversations with patients about vaccines, to reduce hesitancy and increase coverage among pregnant women in all racial and ethnic groups and thereby reduce disparities.^¶¶¶^ CDC has resources to assist providers in effectively communicating the importance of vaccination, such as sharing specific reasons that recommended vaccines are right for the patient and highlighting positive personal or clinical experiences with vaccines.**** In addition, the American College of Obstetricians and Gynecologists has an immunization tool kit^††††^ that includes communication strategies for providers.

### Limitations

The findings in this report are subject to at least five limitations. First, this was a nonprobability sample, and results might not be generalizable to all pregnant women in the United States. Second, vaccination status was self-reported and might be subject to recall or social desirability bias. Third, because of small sample sizes, vaccination coverage could not be assessed separately among some racial and ethnic groups. Fourth, Tdap vaccination coverage estimates might be subject to uncertainty, given the small sample size and exclusion of almost 13% of women whose Tdap vaccination status was unknown. A previous sensitivity analysis showed that actual Tdap vaccination coverage could be 6–7 percentage points higher or lower (*4*). Finally, statistical tests based on the assumption of probability were used to ascertain differences in vaccination coverage among groups in this nonprobability sample and results should be interpreted with caution. Despite these limitations, Internet panel surveys are a useful assessment tool for timely evaluation of influenza, Tdap, and COVID-19 vaccination coverage among pregnant women.

### Implications For Public Health Practice

Maternal vaccination coverage remains suboptimal. Culturally relevant vaccination recommendations from health care providers are critical to improving vaccination coverage, decreasing persistent disparities in vaccination coverage, combatting increases in vaccine hesitancy observed since the start of the COVID-19 pandemic, and reducing adverse maternal and infant illness and associated complications including death from these three vaccine-preventable diseases.

## Footnotes

*
https://www.cdc.gov/coronavirus/2019-ncov/vaccines/stay-up-to-date.html

†
https://www.cdc.gov/coronavirus/2019-ncov/vaccines/recommendations/pregnancy.html

§ Pregnant women were recruited from a large, preexisting, opt-in Internet panel of the general population, operated by Dynata. https://www.dynata.com

¶ A survey response rate requires specification of the denominator at each stage of sampling. During recruitment of an online opt-in survey sample, such as the Internet panels described in this report, these numbers are not available; therefore, a response rate cannot be calculated. Instead, the survey completion rate is provided.

** A woman was considered to have received ≥1 dose of a COVID-19 vaccine if she responded “yes” to the following question, “Have you received at least one dose of a COVID-19 vaccine?”

†† Completion of primary series was assessed through two questions on number of doses and brand of the first or only dose. If a woman reported receiving 2 doses of the Moderna, Pfizer-BioNTech, Novavax, or other vaccines that require 2 doses, or a single dose of the Janssen (Johnson & Johnson) vaccine, she was considered to have competed the primary series. For 66 women who reported being immunocompromised (immunocompromised state from solid organ transplant or blood or bone marrow transplant, immune deficiencies, HIV, use of corticosteroids, or use of other immune weakening medicines), an additional dose was required.

§§ Receipt of a bivalent booster dose was assessed through the follow-up question, “Have you received a bivalent booster vaccine?” which was asked of women who responded “yes” to the question, “Have you received at least one dose of a COVID-19 vaccine?”

¶¶ 45 C.F.R. part 46; 21 C.F.R. part 56; 42 U.S.C. Sect. 241(d); 5 U.S.C. Sect. 552a; 44 U.S.C. Sect. 3501 et seq.

***
https://www.cdc.gov/flu/fluvaxview/dashboard/vaccination-coverage-pregnant.html

†††
https://www.cdc.gov/coronavirus/2019-ncov/need-extra-precautions/pregnant-people.html

§§§
https://www.cdc.gov/flu/fluvaxview/pregnant-women-sept2023.htm

¶¶¶
https://www.thecommunityguide.org/topics/vaccination.html

****
https://www.cdc.gov/vaccines/hcp/adults/for-practice/standards/recommend.html

††††
https://www.acog.org/programs/immunization-for-women/physician-tools
